# At 75: Coming of Aging with GSA

**DOI:** 10.1093/geroni/igaa057.3189

**Published:** 2020-12-16

**Authors:** Jan Abushakrah

**Affiliations:** Portland Community College, Portland, Oregon, United States

## Abstract

This lecture reflects on the highlights of my journey to become a gerontologist: •Joining AGHE in 1998 as PCC explored the development of the first and only Gerontology associate degree in Oregon •Collaborating with community and academic partners to create an applied and evolving curriculum •Empowering older, encore students to translate their experience and compassion into professional careers that embodied their passion •Mentoring hundreds of students to harness their creativity and engage in bold innovation to transform aging lives •Grappling with how to measure and ensure student learning in a way that would make a difference in aging lives •Participating in the working group that developed the Gerontology Education Competencies and currently serving on the founding Board of Governors of the Accreditation for Gerontology Education Council.

